# Supplementary material for: Patterns of Intron Gain and Loss in Fungi
Source: PLoS Biol. 2004 Nov 30;2(12):e422. doi: 10.1371/journal.pbio.0020422 (PMC532390; doi:10.1371/journal.pbio.0020422)
Supplement: Table S1 — Also available at http://genes.mit.edu/NielsenEtAl/. (4.3 MB ZIP). [file pbio.0020422.st001.zip › NielsenEtAl/html/682.html]

AN4386.1.NCU04259.1.MG00957.1.FG09938.1


```
 CLUSTAL W (1.82) Multiple Sequence Alignments - Introns Inserted


Sequence 1: NCU04259.1	696 aa
Sequence 2: FG09938.1	579 aa
Sequence 3: MG00957.1	675 aa
Sequence 4: AN4386.1	637 aa
Alignment Length: 712 aa
Number Identitical Residues: 239 aa
Alignment Score (without introns) 12179


MG00957.1 	MTYKRK2VSLRGVGYLGGMKLNTALGTAASLGRLRPRIGPSITAPSVIQWTCASCRSQ--
NCU04259.1	--MPAQ~TSFPSLAALR--RLQTLNHVYAPLRRTG-------TGRITNGWTCASCRSQLS
FG09938.1 	------~-----------------------------------------------------
AN4386.1  	-MSGMR~VALRTPLRALGDPASVLQYTASPY-----------APRELATWNSYK------
          	        :        .   .      :.            :       .. .      

MG00957.1 	-SSVAGARAAAGFAVGNG~GRRTVVSSIRSITSSRYYR---HASTTSQSQSRSEYQQQQQ
NCU04259.1	ASSASSSSSSSSLSVSLY~NSTLARRSFSSSSKRSSDQNSNGSSSSSSSSSSSNGNGKTN
FG09938.1 	-----------MFSAVLL2GQLLRGKPLQSITSRN------------------FASGKGS
AN4386.1  	-PPRYWLFSSLRFNKATF~PQHNFRTPFSTKTLYRFET-----------QQRAGGQGRQS
          	 ..     ::  :             .: : :                 .. :  . : .

MG00957.1 	QQGGSRRPRGSGALLLATTG-GAASVGILAFTDDIKAGYETVER~CGRVGLALAVCIND2
NCU04259.1	NNNNNNNNNGRRTVLLAAGG-GAAAAGLLAVGDDVKYTYEAVER~TGRVASTLAICVND~
FG09938.1 	RGSNGRKSGPRRPLLYASAGVGTIGATALALGDDIKNSYEAAER1IWRKG-YRTDGLHS~
AN4386.1  	QNSQSRN----KWIGYGIIG-GTIVVGGVVVSDDIRHIYHAAAR~TGRVVGTLAVCIND2
          	. . ...      :  .  * *:  .  :.. **::  *.:. *   *     :  ::. 

MG00957.1 	YRSTLKKRDGLEDGEEQEKLLSECHKRCAVRTLK0SGLKDGKQLADISDHQ~SAMNYLLP
NCU04259.1	YRVTLNAREKIEDPEEKQRLLRECHQRCADRTLE~VLEKSGGIFIKLGQHL0SAMNYLLP
FG09938.1 	YRTTLNAREATEDHDEQENMLKACHKRCAERTLV~VLEKNGGIFIKLGQHL0SAMNYLLP
AN4386.1  	YRVTLKQETCT--PEERNEAIRACHKRCADRTLH~VLERNGSIFIKLGQHL~SSMGYLLP
          	** **: .      :*::. :  **:*** ***     :.*  : .:.:*  *:*.****

MG00957.1 	VEWTTTFIPLQDRCPVSSFESIQAMYRADTGNELWDDFSEFSSEPIGAASLAQVHLATLK
NCU04259.1	PEWTTTFIPLQDKCPVSSFESIERMFLEDTGTSLWDYFSEFSREPIGAASLAQVHLATIK
FG09938.1 	SEWTNTFIPLQDKCPVSSLESIEDMFRQDTGEELWDYFSDFASEPIGAASLAQVHLATIK
AN4386.1  	LEWTTTFIPLQDKCPVSSVESIEQMFVKDTGKRIDELFTTFEPTPIGAASLAQVHIGTLK
          	 ***.*******:*****.***: *:  ***  : : *: *   ***********:.*:*

MG00957.1 	ADGRRVAVKVQHPGLAQWAPLDLALTRYTFQTLKTFFPEYDLSWLSDEMDLSLPQELDFR
NCU04259.1	ETGQRVAVKVQHPSLQRWAPLDMRLTSTTFKTLKYFFPEYDLEWLSSEVEISLPKELDFT
FG09938.1 	GSDQKVAVKVQHPELQGWAPLDLALTRYTFSTLKRFFPEYDLEWLSSEMDVSLPKELDFQ
AN4386.1  	ETGEKVAVKVQHPALAEWAPLDLALTRFTFSMLKRFFPEYDLEWLSKEMDLSLPQELDFR
          	  ..:******** *  *****: **  **. ** *******.***.*:::***:**** 

MG00957.1 	QEAHNANRTREYFSAHP-ELPLIIPDV~IKADRRILIMANESGHRLDDLEYLDGQGIDRD
NCU04259.1	CEAENARRTSRYFAEFAPSLPLVIPDV~LWAKKRLLVMACESGHRLDDLAYMDAYGIDRD
FG09938.1 	EEADNARRMKEHFAKIP-QLPLIIPEV~IWAKQRIIVMACEAGSRLDDLEYMDKNGIDRD
AN4386.1  	MEAQNAKRASEYFKKNS-NAPLVIPKV1LWAQKRILVMEFISGSRPDDLEYLDKNHIDRD
          	 **.**.*  .:*   . . **:**.* : *.:*:::*   :* * *** *:*   ****

MG00957.1 	EVSAALARIFNEMIFGDGAPLHCDPHGGNIAIRKREGGRGVSGGGSNFEVILYDHGLYRD
NCU04259.1	EVSATLARIFNEMIFGEGAPLHCDPHGGNIAIRYHDNSNKS-KSKSNFDIILYDHGLYRD
FG09938.1 	EVSATLSRIFNEMIFGDGAPLHCDPHGGNIAIRKNTSRRGL-GRGPNFDVILYDHGLYRD
AN4386.1  	EVSAALAHIFNEMIFGDNAPLHCDPHGGNIAIRKNPN-----RSRHNFDIILYDHGLYRD
          	****:*::********:.*************** . .         **::**********

MG00957.1 	IPLDLRRSYAKMWLAVVEGDMDRMRKYAHEVAGVTDESFPLFASAITGRDFGVLASSSSG
NCU04259.1	IPLPLRRSYAKLWLAIIDGDIPKMKRYVHEVAGIGEDKFPLFASAITGRDFINVVSAT--
FG09938.1 	IELPMRRSYAKMWLAVIDGDMDRMKKYAHEVAGIEDKDFPLFASAITGRDYS-IVSK---
AN4386.1  	IPRETRRNYAKLWLSVIEADEARMREYSRKVAGVTDEQFPLFASAITGRDYTVLTKKN--
          	*    **.***:**::::.*  :*:.* ::***: :..************:  :.. .  

MG00957.1 	GDGSVLLAPRSLDEKQTMGSALQGGLLADLVQLLGKVPRIILLILKTNDLT~RSLDENLH
NCU04259.1	-DSGGVLKPKEASEQKSMSTALQEGLIVDLVQMLGQVPRIILLILKTNDLT~RALDESLH
FG09938.1 	--SGSILENRTADEQKTMSGALQEGLIVDLVQLLSRVPRIILLILKTNDLT~RSLDENLQ
AN4386.1  	-----ITSLRTEAEKESITGALGEGLLQQLVELLGQVPRIMLLILKTNDLT1RSLDENLH
          	     :   :   *::::  **  **: :**::*.:****:********** *:***.*:

MG00957.1 	TRQGPVRSFLILARYCTRTVFLERLETLRERGS~LFWPPNAVGLLAAWVGYLKVEVQLEA
NCU04259.1	TKQGPVRQFLILARYCMRTVFYEQLEEIRGLGS~IWSPANLVRLVGAWWGMARVEVKLEV
FG09938.1 	TRQGPIRSFMILARYCTKTVFHEKLEEISQNGS~LLWPLNAVRLIAAWVGFMRVEIKLEA
AN4386.1  	TRQGPVRTFLILARYATRTVFEEQMDTINE-SE1FQFLEQPDRDNMYLSGLYLMRNTFDL
          	*:***:* *:*****. :*** *::: :   .. :    :         *   :.  :: 

MG00957.1 	FEMWLSVKRCVVSPVLELVRRSRREEGAVVAL----------------------------
NCU04259.1	FELWLRVKRTLGLGSGFGMGMGTGEEAQQQVVPVGKKGQKRLEREVEAAAAVSVAAAAAA
FG09938.1 	FELWLFTKRMLGMGN----GEFSGAAFQK-------------------------------
AN4386.1  	VPLWLAHLKSTPPANPSRQLQQNHLSITTFFIDQHHD-----------------------
          	. :**   :                           .                       

MG00957.1 	-
NCU04259.1	A
FG09938.1 	-
AN4386.1  	-
          	
```
